# Preparation of Thin-Film Composite Nanofiltration Membranes Doped with N- and Cl-Functionalized Graphene Oxide for Water Desalination

**DOI:** 10.3390/polym13101637

**Published:** 2021-05-18

**Authors:** Francisco J. García-Picazo, Sergio Pérez-Sicairos, Gustavo A. Fimbres-Weihs, Shui W. Lin, Moisés I. Salazar-Gastélum, Balter Trujillo-Navarrete

**Affiliations:** 1 Departamento de Ingeniería Eléctrica y Electrónica, Tecnológico Nacional de México/Instituto Tecnológico de Tijuana, Tijuana, B.C. CP. 22500, Mexico; javier.garciap18@tectijuana.edu.mx (F.J.G.-P.); moises.salazar@tectijuana.edu.mx (M.I.S.-G.); balter.trujillo@tectijuana.edu.mx (B.T.-N.); 2 Centro de Graduados e Investigación en Química, Tecnológico Nacional de México/Instituto Tecnológico de Tijuana, Tijuana, B.C. CP. 22500, Mexico; shuiwhailin@tecijuana.edu.mx; 3 School of Chemical and Biomolecular Engineering, The University of Sydney, Sydney, NSW 2006, Australia; gustavo.fimbresweihs@sydney.edu.au

**Keywords:** graphene oxide, N- and Cl-functionalized graphene oxide, thin-film nanocomposite membrane, interfacial polymerization, nanofiltration

## Abstract

In the present work, chemically modified graphene oxide (GO) was incorporated as a crosslinking agent into thin-film composite (TFC) nanofiltration (NF) membranes for water desalination applications, which were prepared by the interfacial polymerization (IP) method, where the monomers were piperazine (PIP) and trimesoyl chloride (TMC). GO was functionalized with monomer-containing groups to promote covalent interactions with the polymeric film. The composite GO/polyamide (PA) was prepared by incorporating amine and acyl chloride groups into the structure of GO and then adding these chemical modified nanomaterial during IP. The effect of functionalized GO on membrane properties and performance was investigated. Chemical composition and surface morphology of the prepared GO and membranes were analyzed by thermogravimetric analysis (TGA), Raman spectroscopy, FTIR spectroscopy, X-ray photoelectron spectroscopy (XPS), X-ray diffraction (XRD), scanning electron microscopy (SEM), atomic force microscopy (AFM), and transmission electron microscopy (TEM). The fabricated composite membranes exhibited a significant increase in permeance (from 1.12 to 1.93 L m^−2^ h^−1^ bar^−1^) and salt rejection for Na_2_SO_4_ (from 95.9 to 98.9%) and NaCl (from 46.2 to 61.7%) at 2000 ppm, when compared to non-modified membranes. The amine- and acyl chloride-functionalized GO showed improved dispersibility in the respective phase.

## 1. Introduction

Water covers almost 75% of the earth’s surface; nevertheless, it is expected that by 2025 half of the population will live in regions with water shortage [1]. Wastewater treatment and seawater desalination have been proposed to satisfy the increasing demand of this vital resource. Membrane separation processes emerged as a sustainable alternative to provide water to low rainfall areas [2]. Nanofiltration (NF) is a pressure-driven separation process with features similar to those of reverse osmosis (RO). These NF processes are able to partially reject small ions from water such as NaCl, Na_2_SO_4_, CaSO_4_, and CaCl_2_; hence, it can be employed for water desalination [3,4]. Although NF membranes are very commercially successful, there are some major challenges to their performance such as the trade-off effect between salt rejection and water permeability [5,6,7], chlorine resistance [8], and fouling resistance [9].

NF/RO membranes are typically fabricated by the interfacial polymerization (IP) method, which consists of a polycondensation reaction between two monomers occurring at the interface of two non-miscible solutions [10]. Typically, this process begins by wetting a polysulfone porous support with an aqueous amine solution and then applying an organic phase containing the acid chloride monomer; thereby, an ultrathin layer of polyamide (PA) is formed at the interface between the two phases and remains attached to the support [11,12,13]. This PA layer becomes the active layer of the NF membrane since it is exposed to the solutions during separation processes. Thus, many recent studies focus on modifying the PA layer structure [14]. IP enables the formation of a high-density polyamide layer on the support surface. In order to tune the properties of the polyamide layer, several factors may be controlled (e.g., an application method of the monomers [13], the thickness [15], the structure of the monomers [16], the number of layers [17,18], or the incorporation of nanomaterials in the matrix [19]).

Nanomaterials have attracted interest in recent years due to their labile properties. The embedding of nanoparticles into the polyamide layer may increase the hydrophilicity of the membrane and enhance salt rejection performance. Inorganic additives such as titanium dioxide [20,21,22], silica [23], silver [24], zinc oxide [25], and carbon nanotubes [26,27,28,29] have contributed to improving separation performance and increasing the fouling resistance of membranes. Recently, many investigations have studied the effect of the physical immobilization of graphene [30] and graphene oxide [31,32,33] into the polymeric matrix; however, the physical addition of GO in the PA layer produces disaggregation. One strategy is to functionalize GO in order to promote covalent bonding between GO and the polymeric film [34]. The introduction of monomers used during IP on the GO structure may increase the stability and crosslinking of the resulting composite film. The amine and acyl chloride groups may play a role as anchor-like sites for a PA layer to form. Nevertheless, the chemical immobilization of GO in the PA layer and the role of GO during IP have not been extensively discussed.

Graphene is a single layer of honeycomb-like atomic sp^2^ carbon atoms, while graphene oxide (GO) is a highly hydrophilic form of graphene containing bonded functional groups, such as carbonyl, hydroxyl, and epoxide, among others [35]. GO is mainly obtained by chemical exfoliation of bulk graphite using strong oxidant agents [36]. Adding this oxygenated nanomaterial may reduce the surface charge of membranes, thereby increasing electrostatic interactions between solutes and the surface of the membrane [37]. In addition, these aforementioned interactions between the membrane and solutes may reduce concentration polarization, increasing water flux [38]. Another highlight of GO is its capacity to generate (in the presence of water) reactive oxygen species (ROS), which are associated with bacterial growth inhibition, reducing biofouling of NF membranes [39]. These hydrophilic and antimicrobial properties of GO make its application on the polyamide layer very attractive for water flux enhancement. Despite these attributes, the low dispersibility of GO in the phases for interfacial polymerization remains inconvenient.

An innovative approach is to synthesize a functionalized GO monomer for the further incorporation during NF membrane preparation. The presence of amine and acyl chloride groups in GO may, in advance, improve dispersibility in the phases for membrane preparation and yield to covalent bonds between GO and monomers in solution, leading to a more dense film structure. Inducing these nitrogen- and chlorine-containing groups may increase the biofouling resistance of the NF membranes. A similar approach has been reported by Shao et al. [40] and Wen et al. [41].

The aim of this work is to study the effect of different functionalizations of GO on the properties of NF membranes. In this paper, the chemical modification of GO with amine and acyl chloride groups is reported. Both monomer-contained functionalized GO materials were incorporated into monomer solutions to obtain NF membranes, where GO could perform an active role during IP, modifying the characteristics and performance of NF membranes. To evaluate the possible participation of functionalized GO in IP, a reaction was carried out adding only a functionalized GO material and a monomer. The obtained NF membranes were characterized by several microscopic and spectroscopic techniques. The effect of the incorporation of modified GO on membrane performance was further studied. Additionally, the characterization and evaluation of the as-prepared membranes were performed.

## 2. Experimental Section

### 2.1. Reagents

Graphite powder (99.99%), H_2_SO_4_ (98%), ethylenediamine (99%), 1-[Bis(dimethylamino)methylene]-1H-1,2,3-triazolo[4,5-b]pyridinium 3-oxide hexafluoro-phosphate (HATU; 97%), triphosgene (BTC; 98%), carbon tetrachloride (CCl_4_; 99.9%), triethylamine (99.0%), *N*-metil-2-pirrolidone (NMP; 99.5%), 2,2,4-trimethylpentane (iso-octane; 99.8%), piperazine (PIP; 99%), trimesoyl chloride (98%), and Na_2_SO_4_ (99%) were provided from Sigma Aldrich (Saint Louis, MO, USA). H_3_PO_4_ (86%), KMnO_4_ (99%), H_2_O_2_ (30%), and trichloromethane (99.0%) were purchased from Fermont (Monterrey, Mexico). Ethanol (99.9%) and NaCl (99.9%) were acquired from Fraga Lab (Mocorito Mexico). Polyvinyl alcohol (PVA; 513 g mol^−1^) was supplied by Celanese (Irving, TX, USA). Sucrose (${\left[\alpha \right]}_{D}^{25}$ from +66.3° to +66.8°), xylose (98%), and raffinose pentahydrate (98%) were purchased from Spectrum Chemical (New Brunswick, NJ, USA). Fructose (98%) and NaOH (97%) were acquired from Jalmek Scientific (San Nicolas de los Garza, Mexico). Nitrogen gas (99.99%) was purchased from Infra Gases (Naucalpan, Mexico). All reagents were used as received. The aqueous solutions were prepared with DI water (Merck, Milli-Q grade 18 MΩ; Darmstadt, Germany).

### 2.2. Analytical Techniques

Raw and functionalized GO were analyzed by different analytical techniques: thermogravimetric analysis (TGA; TA Instruments, QA-600; New Castle, DE, USA) was carried out from 40–800 °C and 20 °C min^−1^; FTIR spectroscopy (Perkin Elmer, Spectrum 400; Waltham, MA, USA) was performed at a working range of 4000–600 cm^−1^, using ATR device, a resolution of 2 cm^−1^ and 16 scans; Raman spectroscopy (Thermo Scientific, Smart Raman DXR; Waltham, MA, USA) was performed using a range of 800–2200 cm^−1^ and a laser source of 780 nm; X-ray Diffraction (XRD; Bruker, DaVinci; Billerica, MA, USA) was equipped with a source of Cu (λ = 1.542 Å) and a step size of 0.015°; XPS analysis (SPECS^®^ spectrometer with a PHOIBOS^®^ 150 WAL hemispherical energy analyzer) was carried out using an X-ray Al anode (monochromatized) at high intensity mode and a pressure below 1 × 10^−9^ Torr, and high-resolution analysis was taken at a pass energy of 1 eV. XPS was also employed to ensure the chemical functionalization of GO. Deconvolution of the C 1s, N 1s, and Cl 2p peaks in XPS spectra was performed using a Gaussian multipeak fit in Origin 2018 software (version 95e). The C:O atomic ratio was calculated as a ratio of peak intensity for each element. Transmission electron microscopy (TEM; JEOL JEM-2200FS; Akishima, Japan) in STEM mode at 200 kV was performed in order to elucidate GO morphology. For each prepared GO, a selected micrograph was processed by fast Fourier transform (FFT) in ImageJ software (version 1.52a) to determine *d*‑spacing. GO and membrane samples were imaged by SEM (Tescan, Vega 3; Brno, Czech Republic) operated at 20 kV, and a dried sample was placed on double-sided carbon tape. Atomic force microscopy (AFM; NanoSurf, EasyScan 2, Liestal, Switzerland) at contact mode and a scan rate of 1.0–2.0 kHz was employed to determine the surface roughness of prepared membranes. Cross sectional samples were prepared via fracturing in liquid nitrogen and sputtered with Ag (SPI-Module, Sputter Coater; West Chester, PA, USA), and layer thickness was measured using ImageJ.

### 2.3. Preparation and Functionalization of GO

GO was prepared from graphite powder using the Marcano-Tour method with minor modifications [42]. Three grams of graphite was poured into 75 mL of a 9:1 mixture of concentrated H_2_SO_4_ and H_3_PO_4_ at 0 °C (cooling bath) and left under magnetic stirring for 30 min. After that, 1 g of KMnO_4_ was added every 10 min until 6 g was reached. The mixture was kept under stirring at 0 °C for another 30 min, and the cooling bath was then removed. When the mixture reached 35 °C, it was poured into 420 mL of DI water at 4 °C and then heated to 90 °C for 30 min. Afterward, 9 mL of H_2_O_2_ was added into the mixture in order to remove any excess of unreacted KMnO_4_. At this point, the solution turned from dark violet to yellowish brown. The product was cooled to room temperature and left to sit for 8 h. The supernatant was discarded, and the precipitate was rinsed thoroughly with DI water and centrifuged at 8000 rpm for 10 min. This last step was repeated until the pH of the rinse water reached a value of 6. In order to remove any excess of moisture, the product was rinsed with acetone. The product was dried at 50 °C overnight and stored in vacuo. For N-GO preparation, 400 mg of GO was added to 40 mL of ethylenediamine and left under sonication for 20 min at 20 °C, followed by an addition of 4 mg of HATU. The mixture was poured into a 100 mL Teflon autoclave to react solvothermally at 200 °C for 10 h [43]. Once the reaction was completed, the precipitate was rinsed with DI water. The product was collected and dried at 50 °C overnight. To obtain Cl-GO, 3 g of BTC was dissolved in 4 mL of CCl_4_ (BTC/CCl_4_), 50 mg of GO was dispersed in 3 mL of ethanol (GO/ethanol). Both BTC/CCl_4_ and GO/ethanol were mixed in a 50 mL rounded-bottom flask under an ice bath and N_2_ protection. Once the mixture reached 0 °C, a solution of 0.6 mL of triethylamine dissolved in 2 mL of CCl_4_ was added drop-wise until the cloudiness was completely dispersed. The mixture was kept under reflux at 65 °C for 12 h [41,44]. Once the reaction was completed, 40 mL of dry trichloromethane was added and the mixture was left to sit at room temperature. The supernatant was discarded and another four washes proceeded to eliminate any residual by-products from the mixture. All washes proceeded keeping N_2_ gas flow. The solid was finally dried running N_2_ gas and transferred to a tight seal container for further use.

To analyze the potential interactions of functionalized GO with the polyamide layer, an ex situ IP reaction was performed. For this, an aqueous dispersion of N-GO 0.004% was prepared and poured onto a small borosilicate plate, and a drop of a TMC solution at 1% *w*/*v* in iso-octane was then added on top of the plate. Then, we performed a reaction by wetting a borosilicate plate with an aqueous solution of 0.25% *w*/*v* PIP and adding a dispersion of Cl-GO 0.004% *w/v* in iso-octane. A control IP reaction was set using PIP and TMC as monomers in the aqueous and organic solutions, respectively. The purpose of this test was to prove the interactions between N-GO and Cl-GO with the complementary monomer; therefore, it was performed in an isolated environment to eliminate the interference from the PSf substrate.

### 2.4. Fabrication of GO/PA Nanocomposite Membranes

The polysulfone (PSf) support membrane was prepared by casting a solution of 20% *w*/*w* PSf in NMP onto a non-woven fabric (AWA #16) using a knife gap of 170 µm at room temperature. The cast film was immersed into a coagulation water bath for 10 min. The prepared support was rinsed thoroughly using DI water and stored in a water bath at 4 °C. TFC NF membranes were prepared through the IP method. The aqueous phase (0.25% PIP, 0.50% NaOH and 0.25% PVA) was applied onto PSf support membrane using a soft paintbrush, and any excess was then removed with a rubber roller. Next, organic phase (TMC 1% *w*/*v* in dry iso-octane) was poured over an aqueous phase and allowed to react for 30 s. The IP reaction was then stopped by drying with nitrogen gas at 20 L min^−1^ and 3.5 bar for 1 min and then cured at 70 °C for 10 min. Control membrane obtained by IP was labeled as NF1. Next, five different membranes were prepared including nanomaterial (NF2-NF6). Raw GO at 0.004% *w*/*v* was added to NF2 and NF4 in the aqueous and organic phases, respectively. NF3 contains 0.004% *w*/*v* of N-GO in the aqueous phase and NF5 contains Cl-GO 0.004% *w*/*v* in the organic phase. Lastly, NF6 was prepared by adding 0.002% *w*/*v* of N-GO and 0.002% *w*/*v* of Cl-GO into the aqueous and organic phases, respectively. Solution composition for preparation of NF membranes is presented in Table 1.

### 2.5. Performance of NF Membranes

All tests (salt rejection, water flux, and molecular weight cut-off (MWCO)) were evaluated in a cross-flow system as described by Lin et al. [45]. The transmembrane pressure was fixed to 5.5 bar, the effective surface area in the cell was 22.5 cm^2^, and the temperature was set to 25 ± 2 °C. Salt rejection tests were performed using solutions containing either 2000 mg L^−1^ Na_2_SO_4_ or 2000 mg L^−1^ NaCl, while the water flux test was conducted with DI water. Permeate solutions were collected until 10 mL was obtained, and the time was recorded. All measurements were taken twice, and the average value was calculated. Equation (1) was used to determine the permeance (Π) for the membranes (L m^−2^ bar^−1^ h^−1^):

(1) $$\Pi =\frac{V}{A\times P\times t}$$

where *V* is the volume of permeate (L), *A* is the effective area of the membrane (m^2^), *P* is the operating pressure (bar), and *t* is the permeation time (h).

The solute rejection (*R_S_*, %) was computed for each of the membranes according to Equation (2):

(2) $$R_{S}\left(\%\right)=\left(1-\frac{C_{p}}{C_{f}}\right)\times 100$$

where *C_p_* is the concentration of solute in the permeate solution, and *C_f_* is the concentration of solute in the feed solution. For salt rejection tests, salt concentration was determined by measuring the conductivity of the solutions. To determine the MWCO for the prepared membranes, neutral solute rejection tests were carried out using four different saccharide solutions at 1% *w*/*v*: xylose, fructose, sucrose, and raffinose with molecular weights of 150.13, 180.2, 342.3, and 504.5 g mol^−1^, respectively. The concentration of saccharides in the permeate solution was determined by measuring the total organic carbon (TOC; ThermoScientific, HiperTOC analyzer; Waltham, MA, USA). The MWCO was estimated assuming that the membrane pore size distribution behaves log-normally and the fraction of the solute that permeates across the membrane (*θ*) is proportional to the fraction of the membrane pores that are permeable to that solute. This value depends on the Stokes–Einstein radius (*a*). All pores with a pore size greater than *a* will be permeable to the solute. Thereby, *θ*(*a*) is the area under the normalized probability density function (NPDF) of the pore size distribution [46]. *θ*(*z*) is then given by Equation (3).

(3) $$\theta \left(z\right)=\int_{z}^{\infty }\frac{1}{\sqrt{2\pi }}\exp\left(-\frac{z^{2}}{2}\right)dz=\frac{1}{2}\mathrm{erfc}\left(\frac{z}{\sqrt{2}}\right)$$

where *z* is parameterized according to Equation (4):

(4) $$z=\frac{\log\left(a/\bar{a}\right)}{\log\sigma_{a}}$$

Here, *a* is the Stokes radius of the solute, $\bar{a}$ is the geometric mean radius of the pores, and $\sigma_{a}$ is the geometric standard deviation of the pore sizes. By inverting Equation (3) and using Equation (4), we can obtain Equation (5):

(5) $$\log a=\log\bar{a}+\sqrt{2}\log\sigma_{a}{\mathrm{erfc}}^{-1}\left[2\theta \left(z\right)\right]$$

The previous parameter can be evaluated by plotting Equation (5).

The Stokes radius can be estimated as a function of the molecular weight (*MW*) by using Equation (6) [47,48]:

(6) loga=−1.52517+0.47956logMW

## 3. Results and Discussion

### 3.1. Characterization of Functionalized GO

**Figure 1:**
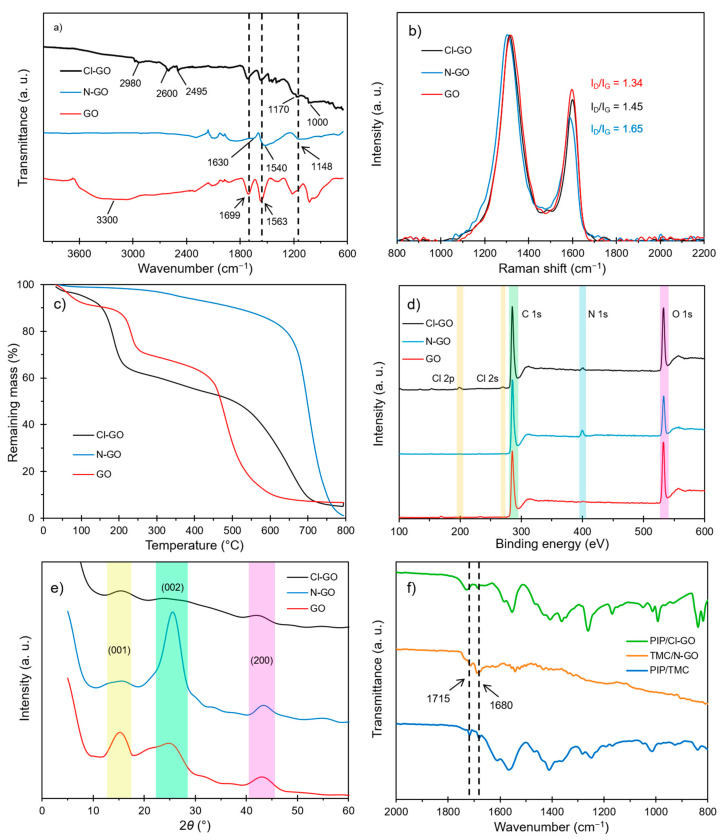
Physicochemical characterization of synthesized GO: (**a**) FTIR spectra, (**b**) Raman spectra, (**c**) TGA curves, (**d**) full XPS spectra, (**e**) XRD diffractograms, and (**f**) FTIR spectra of the ex situ IP reaction.

Results for the physicochemical characterization of synthesized and functionalized GO materials are shown in Figure 1. Firstly, the synthesized GO nanostructures were characterized by FTIR spectroscopy (Figure 1a). The FTIR spectra of pristine GO show intense peaks at 3200–3500, 1699, and 1563 cm^−1^ due to O-H, C=O stretching, and C=C stretching vibrations, respectively [49]. For N-GO, the carboxyl vibration signal is not present, suggesting that, in this case, amine groups displaced the –OH group. Additionally, the C=O stretching peak at 1699 cm^−1^ is no longer present; instead, two peaks appear at 1630 and 1540 cm^−1^, which are related to amide-I and amide-II bands, respectively [40]. Moreover, the weak signal at 1148 cm^−1^ may be related to the C-N bond stretching. The absence of signals for N-H stretching (3500–3700 cm^−1^) may indicate a significant proportion of tertiary amide groups, suggesting that a single ethylenediamine chain may react at different locations in the GO structure. The appearance of signals at 1170, 1000, and 720 cm^−1^ in the FTIR spectra of Cl-GO may be related to C-Cl stretching and asymmetric stretching from carbonyl halide groups [50]. Additionally, the carbonyl peak suffered a slight displacement to a higher wavenumber from 1699 to 1717 cm^−1^, which suggests the presence of acyl chloride functional groups. This last spectrum also shows multiple peaks at 2980, 2600, and 2495 cm^−1^, which may be associated with ammonium salts formed as a byproduct during the synthesis procedure.

The Raman spectrum of prepared GO (Figure 1b) shows a D peak (1340 cm^−1^) and a G peak (1590 cm^−1^); the former peak is associated with carbon lattice distortion, and the latter peak with sp^2^ hybridization of graphitic carbon. A high increase in defect/graphitic band relation (i.e., I_D_/I_G_ ratio) for N-GO and Cl-GO can be observed, when compared to pristine GO. Solvothermal treatment may have induced major structure defects on GO, mostly in C=C bonds, causing the I_D_/I_G_ ratio to increase. Additionally, N and Cl may have been incorporated into the GO structure generating a larger amount of defects.

Thermogram curves of the synthesized materials are presented in Figure 1c. Three major weight losses can be observed for pristine GO. Weight losses at lower temperatures are associated with labile functional groups such as carboxylic acids, so a high degree of functionalization of GO (30%) was obtained. For N-GO, these drops were significantly reduced due to functional group displacement. A drop at 350 °C was present for N-GO instead of at 240 °C for pristine GO, suggesting the successful introduction of more stable functional groups in the GO structure. The increase in thermal stability of N-GO could be related to the presence of amino-carbons and to a partial reduction of GO, as reported by Irani et al. [43]. Cl-GO exhibited a mass drop of 40% at 170–200 °C, which may correspond to acyl chloride groups. This shift to lower temperature may be attributed to the presence of highly labile functional groups in Cl-GO. In accordance with these results, the proposed order for thermal stability of functional groups is NH_2_ > COOH > COCl.

To ensure the attachment of nitrogen- and chlorine-containing functional groups, the chemical composition of prepared GO was analyzed via XPS. As shown in Figure 1d, for pristine GO, only two peaks can be observed, and they are attributed to C 1s (~288 eV) and O 1s (~533 eV) photoelectron lines. N-GO spectra showed a peak at ~400 eV, confirming the presence of N 1s [51]. The C:O atomic ratio increased from 0.883 for pristine GO to 1.264 for N-GO, confirming the de-oxygenation of GO and the introduction of nitrogen. Additionally, XPS spectra of Cl-GO showed two low intensity peaks at 198.5 eV and 271 eV corresponding to Cl 2p_3/2_ and Cl 2s photoelectron lines, respectively [52]. The shift to lower binding energy of Cl 2p_3/2_ transition to 198.5 eV suggests the presence of an alkyl halide in Cl-GO [53]. Likely, Cl displaced O in the GO structure due to a significant increase of the C:O atomic ratio to 1.013. In agreement with the FTIR analysis, the XPS spectrum of Cl-GO shows a low intensity peak at 402 eV, indicating the presence of some residual nitrogen (N 1s); nevertheless, the displacement to higher binding energy suggests that N may have formed ammonium salts, which do not participate in IP [53]. For each material, C 1s peaks were deconvolved into three different peaks at 285, 286.5, and 288 eV, identified as C1, C2, and C3, respectively. C1 corresponded to C bonded with C and H, C2 corresponded to C bonded to electron withdrawing atoms such as O and Cl, and C3 was associated with C=O [53]. Deconvolution of the N 1s peak into N1 (399.5 eV) and N2 (402 eV) was performed for N-GO and Cl-GO. N1 was associated with N bonded to C and H, and N2 was related to N bonded to electron withdrawing atoms (O and Cl) or to N from ammonium salts. From the chemical environment of N bonding, only N1 may participate during IP. An N2 peak was also obtained in order to detect any residual nitrogen in Cl-GO.

Results of the deconvolution for each peak are presented in Table 2 as a fraction of the total amount of the element. It is shown that ~80% of N in N-GO corresponded to N1, which may form covalent interactions with PA. Most of the residual N in Cl-GO belongs to the N2 type, which may not participate in IP.

The crystalline structure of GO-based materials was studied by XRD (Figure 1e). The diffraction pattern of pristine GO showed a characteristic peak at 2*θ* = 14°, associated with the diffraction plane (001), and a second peak at 2*θ* = 25°, corresponding to the plane (002). The weaker and broader peak for (002) suggests that GO is partially reduced [54]. For N-GO, the peak around 2*θ* = 14° decreased; conversely, the peak at 2*θ* = 25° increased, indicating that the amine functionalization process caused a significant reduction of GO, as reported by Irani et al. [43]. This reduction may be related with the higher thermal stability of N-GO as seen in the TGA results. Cl-GO presented a very weak peak at 2*θ* = 25°; thus, the introduction of chlorine may have caused further exfoliation/oxidation of GO, which may have reduced the thermal stability of this material. The results obtained by XRD suggest the successful introduction of functional groups in GO.

The film obtained after the ex situ IP reaction was analyzed using FTIR spectroscopy to study the possible interactions of functionalized GO in the polyamide layer. The results in Figure 1f show that, for the control reaction, there were two peaks at 1715 (I) and 1680 cm^−1^ (II), corresponding to the carbonyl group from carboxyl acid and the amide groups, respectively. When the reaction was performed using only N-GO as the amine-containing monomer, the peak at 1680 cm^−1^ was slightly shifted to 1690 cm^−1^, indicating that the nature of the amide bond is different from that on the PIP/TMC film. The upshifting of the amide signal for TMC/N-GO could be attributed to the formation of a secondary amide or to the presence of amide groups at the solid (i.e., GO nanosheets). It is of note that this signal at 1690 cm^−1^ was not observed in the FTIR spectrum of N-GO; therefore, this signal may come from another source. In the case of the reaction between PIP and Cl-GO as the acyl chloride-containing reactant, both signals (I and II) presented an increase in the wavenumber, which may be because the carboxylic acid and amide groups were at the surface of the GO, which is a more stiff structure.

### 3.2. Morphology Imaging and EDS Characteristics of Functionalized GO

**Figure 2:**
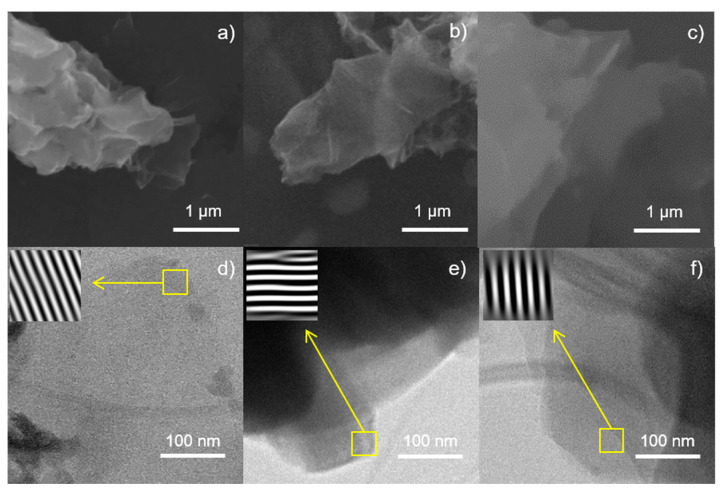
Morphological characterization of synthesized GO. SEM imaging of (**a**) GO, (**b**) N-GO, and (**c**) Cl-GO. TEM bright field imaging of (**d**) GO, (**e**) N-GO, and (**f**) Cl-GO. Inset: magnification of GO layers obtained by FFT processing.

Figure 2 shows the morphology of the prepared GO. SEM characterization showed that N-GO (Figure 2b) and Cl-GO (Figure 2c) present thinner nanosheets, albeit similar in length, compared to the GO (Figure 2a). Micrographs indicate that functionalization occurred at a molecular level and did not affect the overall structure of the GO. In addition, the surface appeared to be smoother for N-GO and Cl-GO than for the pristine GO. This may be because those materials underwent additional steps through synthesis.

Synthesized materials were characterized by TEM to elucidate the effect of functionalizing in GO structure. Introduction of heteroatoms through functionalization with N- and Cl-containing groups, may have caused an increase in the interlayer distance (*d*-spacing). Pristine GO (Figure 2d) showed a *d*‑spacing of 0.79 nm, while those of N-GO (Figure 2e) and Cl-GO (Figure 2f) increased to 0.82 and 0.83 nm, respectively. This lattice distortion suggests that GO was successfully functionalized.

Elemental analysis shows the atomic composition of the prepared nanomaterials (Table 3), confirming the presence of oxygen in GO. N-GO presented a significant increase in the nitrogen atomic ratio, while the chlorine content also increased for Cl-GO. This evidence could indicate that both nitrogen and chlorine were introduced in the GO structure.

### 3.3. Characterization of GO/PA Nanocomposite Membranes

#### 3.3.1. FTIR Spectra of Prepared Membranes

**Figure 3:**
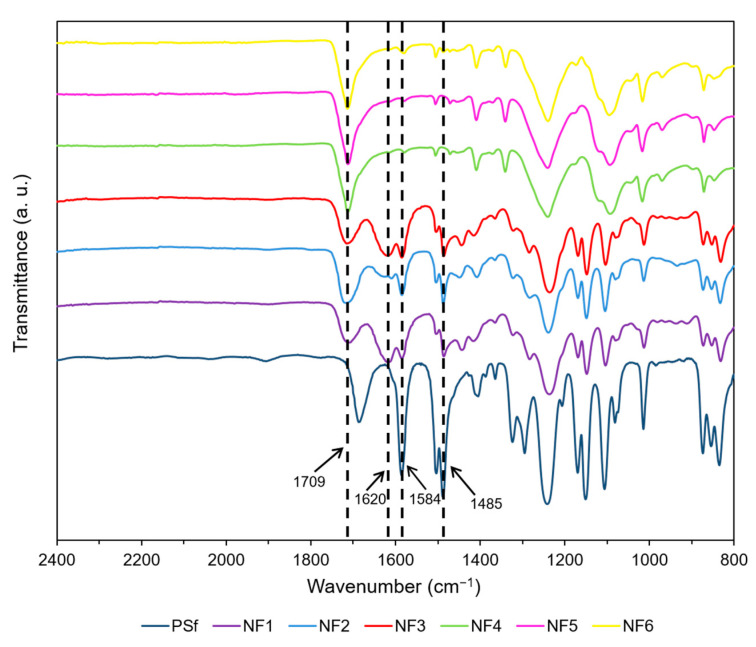
FTIR spectra of prepared membranes.

In order to confirm the incorporation of nanomaterials in the PA layer, the FTIR spectra of the prepared membranes were obtained (Figure 3). As a reference, the spectrum of the PSf support was taken. It is clear that most of the signals in the spectra of NF membranes come from the PSf support. The peaks at 1584 cm^−1^, 1485 cm^−1^, 1220 cm^−1^, and 1120 cm^−1^ correspond to the PSf substrate [55]. The intensity of these signals is lower for the NF membranes since, in these membranes, a PA film covers the PSf. Main differences between NF membranes and PSf are the signals at 1709 cm^−1^ and 1620 cm^−1^. The characteristic peak of the amide I (C=O) stretch at 1620 cm^−1^ appears stronger in membranes NF1, NF2, and NF3 than in membranes NF4, NF5, and NF6 [56]. In addition, the membranes prepared incorporating GO in the organic phase show a stronger carboxylic acid C=O signal at 1709 cm^−1^, indicating the presence of a higher amount of hydrolyzed acyl chloride groups due to solid disaggregation. These results may also be attributed to additional COOH and COCl groups in the GO and Cl-GO, respectively, added during the preparation of membranes with incorporated GO-based material in the organic phase. NF3 shows a weaker carboxylic C=O peak (1709 cm^−1^) than the membrane NF2, which could be related to the less carboxylic groups in N-GO. A stronger amide C=O peak (1620 cm^−1^) can be observed in NF3 compared to NF2, indicating the presence of amine groups in N-GO. Both NF1 (blank) and NF3 showed similar behavior for C=O (1620 cm^−1^) peak intensity, indicating that the addition of N-GO may have enhanced crosslinking, either by preventing disaggregation of the solid or by forming covalent interactions with PA chains.

#### 3.3.2. SEM Imaging of GO/PA NF Membranes

**Figure 4:**
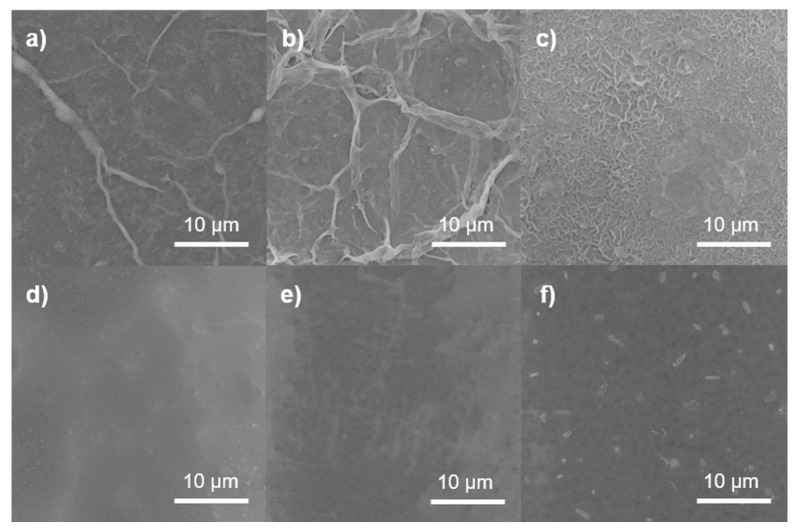
SEM images of NF membranes: (**a**) NF1, (**b**) NF2, (**c**) NF3, (**d**) NF4, (**e**) NF5, and (**f**) NF6.

**Figure 5:**
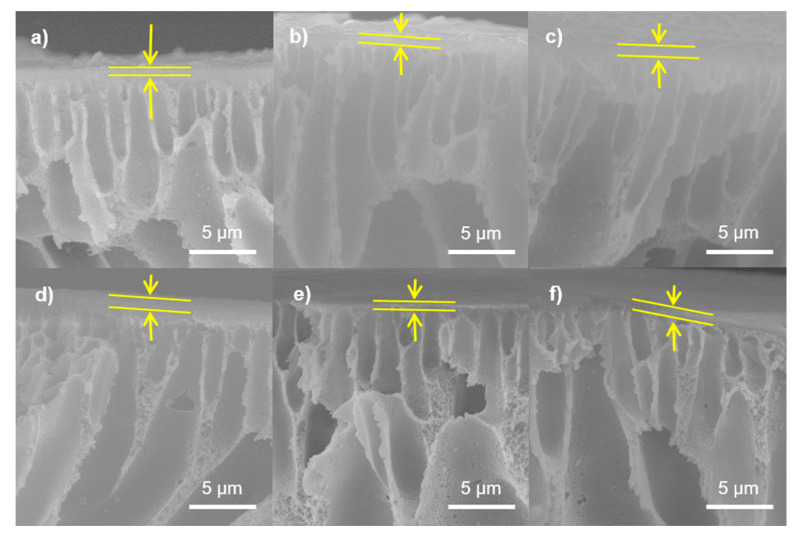
Cross-sectional images obtained by SEM for prepared NF membranes: (**a**) NF1, (**b**) NF2, (**c**) NF3, (**d**) NF4, (**e**) NF5, and (**f**) NF6.

Micrographs obtained by SEM are depicted in Figure 4. NF1 (Figure 4a) shows a very smooth surface, while NF2 (Figure 4b) presents a wrinkled morphology, indicating that GO may have caused the PA layer to become uneven in the latter membrane. A uniform particle distribution with small nodules can be observed in the surface of NF3 (Figure 4c); these agglomerates have diameters smaller than 100 nm. This suggests that the functionalization of GO with amine groups may enhance the degree of crosslinking. In membranes NF4 (Figure 4d) and NF5 (Figure 4e), a granular morphology can be observed, indicating that only some scattered PA chains formed. The presence of the higher-density PA around the Cl-GO particles, as depicted in Figure 4f, may suggest that the GO was successfully functionalized. Figure 5 shows cross-sectional images of the membranes, showing the structure of the porous PSf support and confirming the formation of a top dense-film layer of PA. The blank membrane (NF1) showed a dense layer measuring 218 ± 5 nm in thickness, and for NF2, this was 177 ± 3 nm. This structural change in the membrane surface may indicate that, in NF2, IP may have proceeded mostly around GO nanoparticles. However, N-GO-doped membranes presented an increase in film thickness, indicating that this nanomaterial may have participated in IP. It is important to highlight that, during the preparation of the organic phase for NF4, a non-homogeneous dispersion was observed, implying that, during the preparation of this membrane, some agglomerates of GO were formed on the membrane surface. In addition, SEM imaging suggests the formation of a thicker (254 ± 7 nm) and smoother PA layer. In this case, GO may only have played a role as a scaffold for the formation of polymer chains. Due to an improvement in dispersability, Cl-GO promoted the formation of a thinner layer (197 ± 3 nm).

#### 3.3.3. AFM Imaging of GO/PA NF Membranes

**Figure 6:**
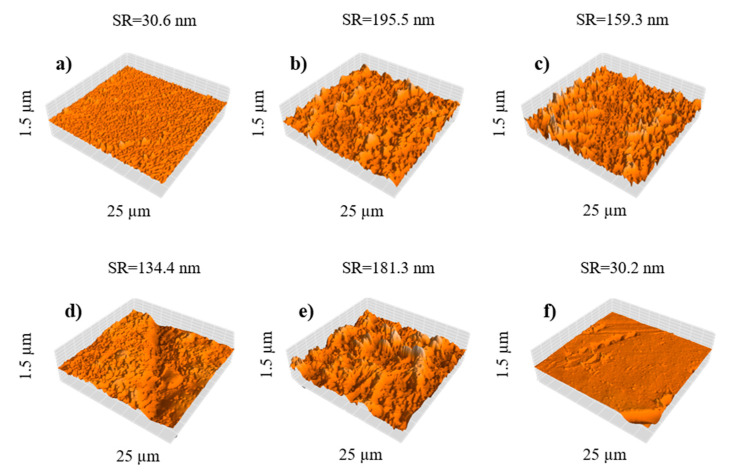
Surface topography of NF membranes: (**a**) NF1, (**b**) NF2, (**c**) NF3, (**d**) NF4, (**e**) NF5, and (**f**) NF6.

For atomic force microscopy characterization, a 25 µm window was analyzed. The resulting micrographs (Figure 6) revealed that membrane surface roughness (SR) increased when pristine GO was added, due to the formation of aggregates in the aqueous phase. This is consistent with previous reports by Sirinupong et al. [57]. Comparing the addition of GO (Figure 6b) and that of N-GO (Figure 6c), it is shown that N-GO can reduce membrane surface roughness from 195.5 to 159.3 nm, as this material is more stable when dispersing into the aqueous phase, avoiding lump formation. AFM showed a higher peak-to-peak distance for the membrane NF4 (Figure 6d), which could be attributed to the low stability of GO in iso-octane. The addition of Cl-GO also increased membrane surface roughness to 181.3 nm (Figure 6e). Since Cl-GO was uniformly dispersed in the organic phase, the increase in surface roughness could be attributed to a thicker PA layer. Surprisingly, NF6 (Figure 6f) exhibited a topography similar to that of the blank membrane (NF1), suggesting that, because of the lower amount added to both aqueous and organic phases, most of the functionalized GO could be located at the interface during IP and thus be embedded at the center of the PA layer. This behavior may indicate that functionalized GO is attracted to the interface due to the reacting functional groups on its structure.

### 3.4. Performance of NF Membranes

#### 3.4.1. Water Flux Test

**Figure 7:**
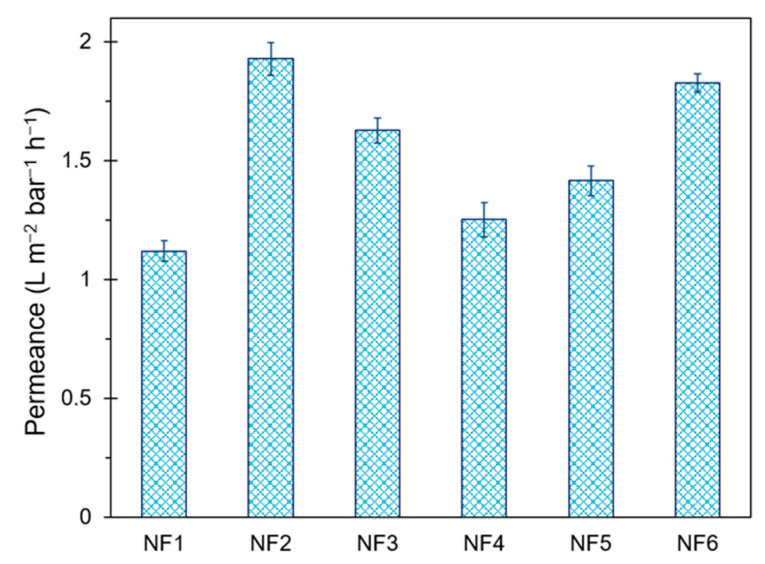
Permeance results in pure water tests for as-prepared membranes.

All performance tests were carried out in the same chronological order to ensure reliable results. Pure water flux results are shown in Figure 7. Permeance increased from 1.120 to 1.929 L m^−2^ bar^−1^ h^−1^ due to the addition of GO during IP. It is likely that GO disrupted the polymer chains, increasing both pore size and hydrophilicity of the membrane surface. A similar behavior was observed for N-GO. Although N-GO presented less functional groups than GO, a slight increase in permeance for NF3 was observed, compared to NF1. This effect may suggest that fewer groups in N-GO confer high hydrophilicity. Membranes prepared with GO-based materials in the organic phase showed significantly less permeance (up to 35% lower compared to NF2); this may be related to the SEM and AFM results, which suggest the presence of a thicker selective layer in NF4 and NF5. On the other hand, NF6 reached almost the same permeance value as NF2, while it exhibited a lower surface roughness than the blank membrane.

#### 3.4.2. Salt Rejection Capacity

**Figure 8:**
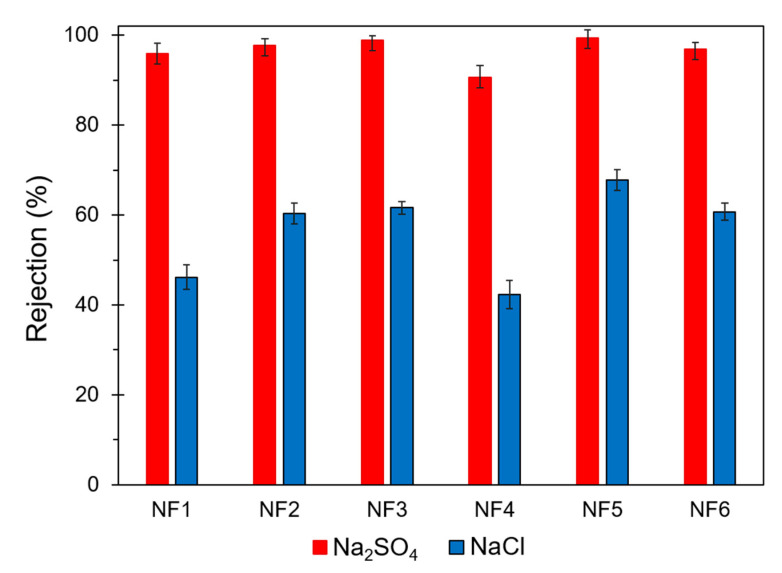
Na_2_SO_4_ and NaCl rejection capacity of prepared NF membranes.

Results from salt rejection tests are presented in Figure 8. All membranes showed a low NaCl rejection capacity, although the membrane NF5 presented the highest rejection (67.8%) for this salt. The membrane NF4 presented the lowest salt rejection and the lowest permeance. This behavior is related to the uneven distribution of the film due to the low dispersability of GO during preparation, as discussed in the SEM section. The formation of these agglomerates may have reduced the filtration effective area for this membrane. Aqueous phase-modified membranes exhibited an increase in Na_2_SO_4_ rejection up to 3% compared to non-modified membrane. Cl-GO membranes presented the highest Na_2_SO_4_ rejection (up to 99.4% for NF5) at the expense of a lower permeance. This trade-off behavior between water flux and salt rejection is usual in NF membranes [58].

#### 3.4.3. Molecular Weight Cut-Off Determination

**Figure 9:**
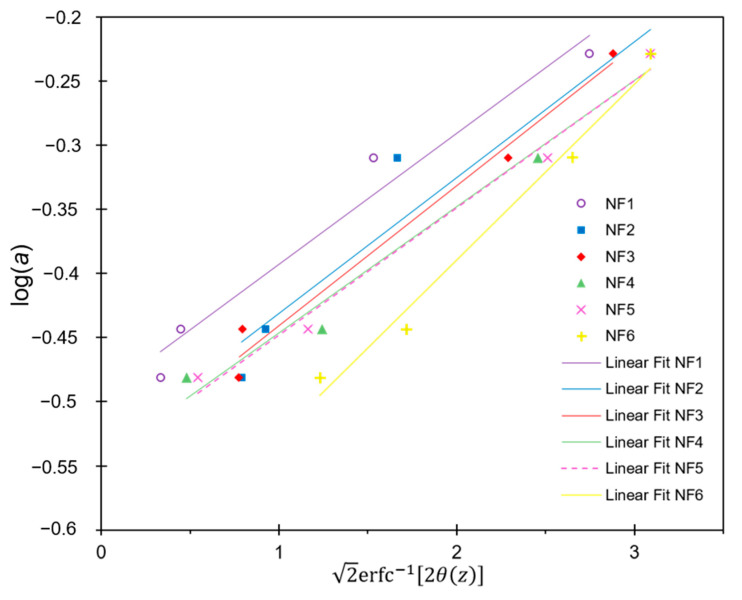
Plot of log (*a*) vs $\sqrt{2}{\mathrm{erfc}}^{-1}\left[2\theta \left(z\right)\right]$ for the prepared NF membranes.

The MWCO for the different membranes was determined by interpolation using Equation (5). The plot of Equation (5) is shown in Figure 9. Results showed that aqueous phase-modified membranes presented lower saccharide rejection, compared to organicphase-modified membranes. This behavior may be caused by a thinner and more porous PA layer in the aqueous phase-modified membranes. The blank membrane presented the highest MWCO (264 g mol^−1^). The NF2 and NF3 membranes achieved outstanding salt rejection without a significant reduction of neutral solute rejection capacity, as they presented an MWCO of 221 and 212 g mol^−1^, respectively. This behavior may indicate that the separation mechanism for NF2 and NF3 is governed by electrostatic interactions rather than by solute size. A higher saccharide retention (MWCO < 203 g mol^−1^) for organic phase-modified membranes suggests the presence of a denser PA layer. This shows agreement with previous results obtained by microscopy.

Table 4 presents a summary of the most important characteristics of the prepared NF membranes.

## 4. Conclusions

**Figure 10:**
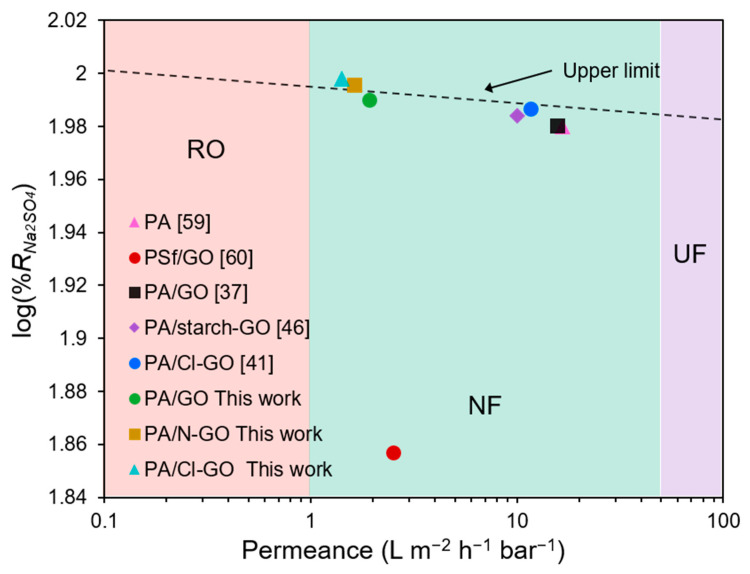
Correlation of permeance and observed Na_2_SO_4_ rejection for similar NF membranes.

A simple method for preparing N- and Cl-functionalized GO was accomplished with a high degree of functionalization (~15% and ~16% for N and Cl, respectively). The dispersability of GO in the organic phase was significantly improved due to the incorporation of COCl groups. The developed ex situ IP reaction proved that it is possible for N-GO and Cl-GO to form amide bonds as expected for PIP and TMC, as confirmed by FTIR. The NF membranes prepared adding GO-based materials in the aqueous phase showed remarkable performance for Na_2_SO_4_ separation, with very similar permeance in comparison to other reported NF, which is evidenced in Figure 10 [37,41,46,59,60]; thereby, further applications for water desalination are feasible. On the other hand, prepared Cl-GO/PA membranes presented significantly less permeance but a higher salt rejection capacity. Cl-GO-added membranes showed an increase in saccharide rejection capacity, making other potential applications such as dye or volatile organic compound removal possible. The enhanced dispersability of functionalized GO materials allows for a more uniform distribution of GO in the PA layer, modifying membrane properties. The incorporation of chemically modified GO in the structure of NF membranes appears to be a suitable strategy for improving its permeance.

## Figures and Tables

**Table 1:** Reagents concentrations in aqueous and organic phases for the prepared membranes (percentage is expressed as *w*/*v*).

	Reagent	NF1	NF2	NF3	NF4	NF5	NF6
	PIP	0.25%	0.25%	0.25%	0.25%	0.25%	0.25%
	NaOH	0.50%	0.50%	0.50%	0.50%	0.50%	0.50%
Aqueous phase	PVA	0.25%	0.25%	0.25%	0.25%	0.25%	0.25%
	GO	-	0.004%	-	-	-	-
	N-GO	-	-	0.004%	-	-	0.002%
	TMC	1.00%	1.00%	1.00%	1.00%	1.00%	1.00%
Organic phase	GO	-	-	-	0.004%	-	-
	Cl-GO	-	-	-	-	0.004%	0.002%

**Table 2:** Relative ratio (%) of atomic species obtained by deconvolution of XPS spectra.

Sample	C1	C2	C3	N1	N2
GO	69.7	5.2	25.1	N/D	N/D
N-GO	62.4	16.8	20.8	78.0	20.0
Cl-GO	68.7	9.8	21.5	24.5	75.5

**Table 3:** Atomic composition of prepared GO obtained by EDS analysis.

Sample	C	O	N	Cl
GO	54%	43%	4%	0%
N-GO	57%	19%	23%	1%
Cl-GO	70%	6%	8%	16%

**Table 4:** Summary of results for the PA nanocomposite membranes.

Membrane	Permeance (L m^−2^ bar^−1^ h^−1^)	Rejection for Na_2_SO_4_ (%)	Rejection for NaCl (%)	MWCO (g mol^−1^)	Surface Roughness (nm)
NF1	1.12 ± 0.04	95.9 ± 2.3	46.2 ± 2.7	264 ± 5	30.6 ± 0.6
NF2	1.93 ± 0.07	97.7 ± 1.6	60.3 ± 2.3	221 ± 8	195.5 ± 3.9
NF3	1.63 ± 0.05	98.9 ± 1.1	61.6 ± 1.4	212 ± 7	159.3 ± 3.2
NF4	1.25 ± 0.07	90.7 ± 2.6	42.3 ± 3.1	203 ± 6	134.4 ± 2.7
NF5	1.42 ± 0.06	99.4 ± 1.8	67.8 ± 2.3	202 ± 8	181.3 ± 3.6
NF6	1.83 ± 0.04	96.9 ± 1.5	60.7 ± 1.9	145 ± 7	30.2 ± 0.6

## Data Availability

The data presented in this study are available on request from the corresponding author.
